# Reappraisal of the Therapeutic Role of Celecoxib in Cholangiocarcinoma

**DOI:** 10.1371/journal.pone.0069928

**Published:** 2013-07-26

**Authors:** Chun-Nan Yeh, Kun-Chun Chiang, Horng-Heng Juang, Jong-Hwei S. Pang, Chung-Shan Yu, Kun-Ju Lin, Ta-Sen Yeh, Yi-Yin Jan

**Affiliations:** 1 General Surgery Department, Chang Gung Memorial Hospital, Kwei-Shan, Taoyuan, Taiwan, R.O.C; 2 General Surgery Department, Chang Gung Memorial Hospital, Keelung, Taiwan, R.O.C; 3 Graduate Institute of Clinical Medical Sciences, College of Medicine, Chang Gung University, Kwei-Shan Tao-Yuan, Taiwan, R.O.C; 4 Department of Anatomy, College of Medicine, Chang Gung University, Kwei-Shan Taoyuan, Taiwan, R.O.C; 5 Institute of Nuclear Engineering and Science, National Tsing-Hua University, Hsinchu, Taiwan, R.O.C; 6 Department of Nuclear Medicine, Chang Gung Memorial Hospital, Kwei-Shan, Taoyuan, Taiwan, R.O.C; Universidade Federal do Rio de Janeiro, Brazil

## Abstract

Cholangiocarcinoma (CCA), a lethal disease, affects many thousands worldwide yearly. Surgical resection provides the best chance for a cure; however, only one-third of CCA patients present with a resectable tumour at the time of diagnosis. Currently, no effective chemotherapy is available for advanced CCA. Cyclooxygenase-2 (COX-2) is a potential oncogene expressing in human CCA tissues and represents a candidate target for treatment; however, COX-2 inhibitors increase the risk of negative cardiovascular events as application for chemoprevention aim. Here, we re-evaluated the effectiveness and safety of celecoxib, one widely used COX-2 inhibitor, in treating CCA. We demonstrated that celecoxib exhibited an anti-proliferative effect on CGCCA cells via cell cycle arrest at G2 phase and apoptosis induction. Treatment for 5 weeks high dose celecoxib (160 mg/kg) significantly repressed thioacetamide-induced CCA tumour growth in rats as monitored by animal positron emission tomography through apoptosis induction. No obviously observable side effects were noted during the therapeutic period. As retrospectively reviewing 78 intrahepatic mass-forming CCA patients, their survival was strongly and negatively associated with a positive resection margin and high COX-2 expression. Based on our result, we concluded that short-term high dose celecoxib may be a promising therapeutic regimen for CCA. Yet its clinical application still needs more studies to prove its safety.

## Introduction

Cholangiocarcinoma (CCA), which originates in the epithelial lining of the biliary tract, is the second most malignant tumour in the liver after hepatocellular carcinoma [1]–[3]. CCA accounts for 10–15% of hepatobiliary neoplasms, and its incidence and mortality have recently increased [4], [5]. The survival rate of patients with CCA is very poor, and surgical resection provides the best chance for a cure [6]–[8]. However, the difficulty in early diagnosis makes most patients poor candidates for surgery. For patients with unresectable CCA, prognosis is dismal and most die within 1 year [9]. Additionally, CCA is resistant to traditional chemotherapy and radiotherapy. Thus, advanced CCA represents a challenge for clinicians, and the establishment of a new therapeutic regimen for CCA is urgent and warranted.

Local inflammation of the biliary tree may be the main cause of epithelial transformation of the biliary tract from dysplasia to subsequent malignancy. Reparative proliferation of the biliary epithelium after wounding, primary sclerosing cholangitis, clonorchiasis, hepatolithiasis, parasitic infestation, and other conditions is associated with increased incidence of CCA [4], [10], [11].

Cyclooxygenase-2 (COX-2), the inducible form of prostaglandin endoperoxidase, is constitutively expressed in some human CCA cell lines [12]. COX-2 also plays an important role in eliciting cholangiocarcinogenesis in human and rat models [13], [14]. Moreover, the overexpression of COX-2 as determined by immunohistopathologic staining of CCA was reported in humans [12], [15], [16]. Thus, COX-2 represents a potential oncogene in CCA, and a COX-2 inhibitor may represent a new treatment for CCA. Previously, the COX-2 inhibitors JTE-522 and NS-398 were shown to inhibit cell growth in 5 human COX-2-expressiing CCA cell lines at concentrations of 100 µM and 200 µM, respectively [12]. Celecoxib, one of the most widely used clinical COX-2 inhibitors, represses rat C611B CCA cell growth markedly in a dose-dependent manner [14]. Further, celecoxib exerted this anti-proliferative effect on CCA in vivo in a xenograft animal model [17]. However, the clinical use of COX-2 inhibitors is hampered because of their potential for causing serious cardiovascular events when applied for chemoprevention [18].

We previously generated a rat CCA cell line (CGCCA) [19] derived from the thioacetamide (TAA)-induced CCA rat model [20], in which CCA can be induced by administering TAA-containing water for 20 weeks. This model successfully recapitulates human CCA progression, and rat CCA tumour growth can be easily evaluated by animal PET [21]. In this study, we aimed to examine the presumed anti-proliferative effect of celecoxib on CCA in vitro and in vivo to re-examine its effectiveness. We also retrospectively reviewed patients with mass-forming CCA (MF-CCA) to investigate the relationship of COX-2 expression with clinical characteristics and prognosis of CCA patients in an effort to re-evaluate the role of COX-2 inhibitors in CCA treatment.

## Materials and Methods

### Cell Culture

The TAA-induced CCA cell line (CGCCA) established previously in our laboratory [19] was grown in a cell culture medium containing Dulbecco’s Modified Eagle Medium with 100 U/ml penicillin and 100 U/ml streptomycin (basal medium) plus 10% foetal bovine serum. The medium was freshly prepared as indicated with a final concentration of 0.1% dimethyl sulfoxide (DMSO) and was changed every 2 days.

### Immunohistochemical Staining of CCA Cells and Rat Tissue for COX-2

CGCCA cells were seeding at 2000 cells/well in a culture slide (#154461 Lab-Tek II Chamber Slide System, Nalge Nunc International, USA) and incubated at 37°C incubator overnight. After rinsed with PBS twice, the cells were fixed with 4% PFA for 1 min and were perforated with 1% tween 20 for 1 min. The slide was prepared as usual method for routine IHC staining. Slides were incubated with primary monoclonal antibody against COX-II (RB-9072-P Lab vision corporation, Fremont, CA, USA, dilution 1∶400) overnight at 4°C. The slides were then washed three times for 5 minutes in TBST before visualization with LSAB2 system-HRP (No K0675 Dako Cytomation, Carpinteria, USA). Control slides were incubated with secondary antibody only.

### BrdU Assay

The BrdU assay was performed using a BrdU ELISA kit (Roche Cell Proliferation ELISA, BrdU (colorimetric) #11647229001, Roche Diagnostrics, GmbH, Mannheim, Germany). Briefly, cells were plated in a 96-well plate, cultured to approximately 50% confluence, and treated with the indicated concentrations of celecoxib for 20 h. The harvest and labelling procedures adhered to those described in the kit manual.

### MTT Assay

Cell viability and proliferation rate were measured using 3-(4,5-dimethylthiazol-2-yl)-2,5-diphenyltetrazolium bromide (MTT) assays (MTT Cell Growth Assay Kit, #CT01, Chemicon, Chemicon International, Temecula, CA, USA). Relative MTT absorbance was measured at 570 nm on a Syva Autotrak EIA Autoreader (Bio-Tek Instruments Inc, Winooski, VT, USA).

### Cell Cycle Analysis by Flow Cytometry

Flow cytometry for cell cycle analysis was performed using a FACSCalibur (BD Biosciences, San Jose, CA, USA) as described previously [22].

### Western Blot

Whole cell lysates of CCA cell lines were obtained using Pierce RIPA buffer (Thermo, USA). Protein samples were separated on 8–12% gradient sodium dodecyl sulphate-polyacrylamide gels and transferred to Immobilon-P membranes (Millipore, Bedford, MA, USA). Antigen-antibody complexes were detected using the ECL blotting analysis system (Millipore). The following primary antibodies were used: CDK1 (Santa Cruz Biotechnology, Santa Cruz, CA, sc-166885), Cdc25C (Santa Cruz Biotechnology, sc-5620), P21 (Thermo Fisher Scientific Inc, Wilmington, DE, USA, #MS-387-P), P27 (Cell Signal, Danvers, MA, USA, #3698), and β-actin (Novus, NB600-501). The protein expression levels were quantified using the open-source software package Image J and normalised to β-actin levels.

### Propidium Iodide (PI) Staining

After treatment with celecoxib at the indicated concentrations, the cultured cells were stained with a solution containing 4 µg/ml PI and 100 µg/ml RNaseA in 1× PBS and incubated in the dark for 30 min.

### Apoptosis Analysis by TUNEL Assay

The TUNEL assay procedure was described previously [23]. Cellular DNA was stained using an apoptosis detection kit (Millipore). Tissues DNA was stained by TumorTACS In Situ Apoptosis Detectin kit (TREVIGEN #4815-30-K). The assay was performed according to the manufacturer’s instructions.

### Animal Studies

All animal studies were approved by the experimental animal ethics committee at Chang Gung Memorial Hospital and conformed to the US National Institute of Health (NIH) guidelines for the care and use of laboratory animals (Publication No. 85–23, revised 1996). A total of 40 adult male Sprague-Dawley (SD) rats with a mean weight of 250±14 g were used in the experiments. The animals were divided into 1 control group (n = 10) and 3 experimental groups (n = 30). The rats were housed in an animal room with a 12/12-hour light-dark cycle (light from 8∶00 AM to 8∶00 PM) at an ambient temperature of 22±1°C. SD rats were administered 300 mg TAA/L daily in their drinking water for up to 20 weeks [20]. Celecoxib in water was administered by gavage to the 3 experimental groups at doses of 40, 80, or 160 mg/kg (10 rats in each group) once daily at between 9∶30 and 10∶00 AM. The 10 rats in the control group received water only according to the same schedule. Treatment was administered for 5 days/week over a 33-day period (from the 20^th^ to the 25^th^ week).

### Efficacy Evaluation with Positron Emission Tomography (PET) Imaging

To evaluate glycolysis alteration in the liver tumours of living animals, all rats underwent 18F-fluorodeoxyglucose (^18^F-FDG) PET studies at the molecular imaging centre of Chang Gung Memorial Hospital. All 40 rats underwent serial PET scans at weeks 20, 21, and 25 after toxin treatment using the Inveon™ system (Siemens Medical Solutions Inc, Malvern, PA, USA). Details regarding radioligand preparation, scanning protocols, and optimal scanning time are described in our previous report [21]. Quantification of ^18^F-FDG uptake in the largest liver tumour and normal liver tissue was performed according to the recommendations of the European Organization for Research and Treatment of Cancer [24] by calculating the standardised uptake value (SUV) using the following formula:
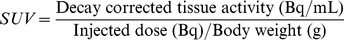



The tumour regions of interest (ROIs) were determined according to the largest diameter of the selected tumour in transverse images, and the ROIs of apparently normal liver tissue were determined from the same transverse images. The tumour and liver mean SUVs (SUV_mean_) and the tumour-to-liver (T/L) radioactivity ratio were calculated for comparisons.

### Apoptosis Determined by DNA Laddering

DNA ladders were analysed using the QIAamp DNA Mini Kit #51304 (QIAGEN, Valencia, CA, USA). Briefly, up to 25 mg of tissue was ground thoroughly in liquid nitrogen with a mortar and pestle. Buffer ATL (containing proteinase K) was added, and the sample was incubated at 56°C until complete lysis was achieved. The RNA was removed using RNase A, and the sample was purified using a QIAamp Mini spin column. The DNA quantity was determined on a TECAN Infinite M200 PRO machine and analysed on a 2% agarose gel.

### Clinicopathological Features of 78 Patients with Intrahepatic MF-CCA

From the archives of Chang Gung Memorial Hospital, 78 MF-CCA patients who had undergone hepatectomy between 1989 and 2006 were selected based on the availability of sufficient quantities of tumour cells. Intrahepatic CCA was defined as carcinoma that arose from distal second order (or higher) branches of the intrahepatic ducts. Curative resection was defined as a negative resection margin observed during histopathological examination. Surgical mortality was defined as death that occurred within 1 month of surgery. Laboratory tests were conducted on the day before the surgery. The tumour stage was defined according to the pathological tumour node metastasis (pTNM) classification proposed by the American Joint Committee on Cancer (AJCC), 6^th^ edition. This retrospective study was approved by the institutional review board at Chang Gung Memorial Hospital (clinical study No. 99–2886B). Written consent was given by the patients for their information to be stored in the hospital database and used for research.

### Statistics

All data are presented as means with standard deviations (SD). Differences between experimental animals and controls were calculated using the Mann-Whitney *U* test or the Kruskal-Wallis test. The overall survival rates were calculated using the Kaplan-Meier method. Sixteen clinicopathological variables were selected for difference analysis by using the log-rank test (univariate). The Cox proportional hazards model was employed for multivariate regression analysis. SPSS statistical software for Windows was used for the statistical analysis (SPSS version 10.0, Chicago, IL, USA). *P*≤0.05 was considered statistically significant.

## Results

### Anti-proliferative Effect of Celecoxib on CGCCA Cells in Vitro

**Figure 1:**
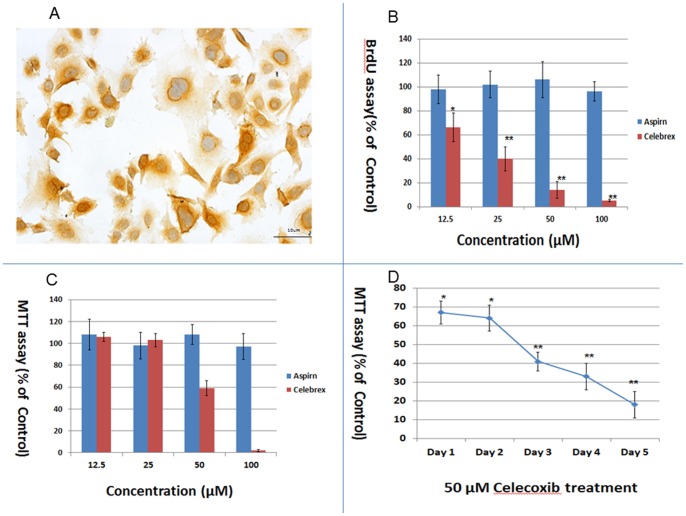
Celecoxib induced a potent anti-proliferative effect on CGCCA cells. (A) CGCCA cells with strong positive cytoplasmic immunoreactivity for COX-2 (400X). (B) Concentration-dependent anti-proliferative effect of celecoxib on CGCCA cells. CGCCA cells were incubated with various concentrations (12.5, 25, 50, and 100 µM) of celecoxib for 24 h. Cell proliferation was determined using the BrdU assay. (C) After 3 days of celecoxib treatment, cell viability was measured by the MTT assay. (D) Celecoxib (50 µM) induced growth repression in CGCCA cells in a time-dependent manner. Results are presented as % of the control. Each value is the mean ± SD of 3 to 5 determinations. *p<0.05, **p<0.001 (versus control).

The CGCCA cell line previously developed in our lab [19] showed prominent cytoplasmic expression of COX-2 as determined by immunohistochemical staining (Figure 1A). Therefore, we applied the selective COX-2 inhibitor celecoxib to investigate the presumed anti-proliferative effect of celecoxib on CGCCA cells. Figure 1B shows that 1-day celecoxib treatment inhibited the proliferation of CGCCA cells in a concentration-dependent manner as determined by the BrdU assay. No inhibitory effect of aspirin on CGCCA cell growth was noted (Figure 1B). As shown in Figure 1C, cultured CGCCA cells were treated with 12, 25, 50, and 100 µM of celecoxib and aspirin, and the MTT assay was performed on day 3. No significant effect was observed in CGCCA cells treated with 12 and 25 µM celecoxib; however, 50 µM of celecoxib induced 41% ±7% growth inhibition. Notably, almost all the cells treated with 100 µM celecoxib for 3 days died. As for aspirin, no cell growth inhibition for CGCCA cells was detected.

We then conducted a time course experiment using 50 µM celecoxib. As shown in Figure 1D, 33% ±6%, 36% ±7%, 59% ±5%, 67% ±7%, and 82% ±7% cell growth inhibition was observed on days 1, 2, 3, 4, and 5, respectively.

Taken together, our results indicate that celecoxib induced concentration- and time-dependent inhibition of CGCCA cell proliferation.

### Cell Cycle Distribution Analysis

**Figure 2:**
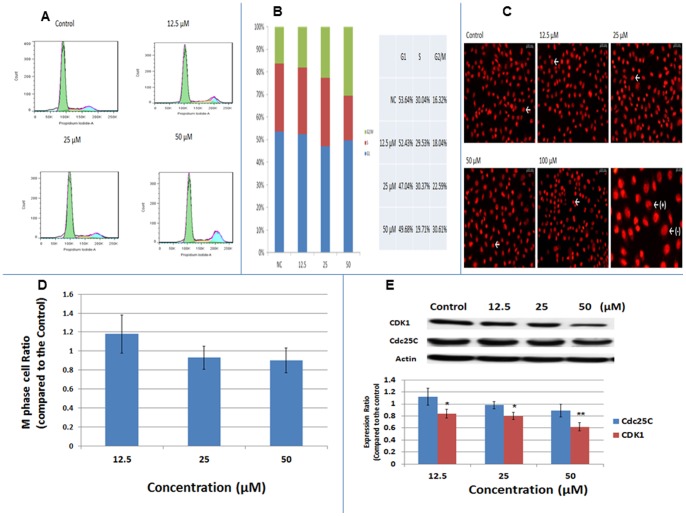
Celecoxib induced cell cycle arrest at the G2 phase in CGCCA cells. CGCCA cells were treated with the indicated concentration of celecoxib for 1 day, and the cell cycle distribution was analysed. (A and B) After 1 day of celecoxib treatment, CGCCA cells were analysed by flow cytometry to determine cell cycle distribution. (C) Treated CGCCA cells were stained with PI to show condensed chromatin (white arrow) in M-phase cells. In the right lower panel, the (+) and (–) are examples of cells with and without condensed chromatin, respectively. (D) Ratio of CGCCA cells at M-phase. (E) Expression levels of Cdc25C and CDK1 in CGCCA cells after 1 day of treatment with the indicated concentrations of celecoxib as measured using western blot. Results are presented as % of the control. Each value is a mean ± SD of 3 to 5 determinations. *p<0.05, **p<0.001 (versus control).

To further understand the mechanism underlying the anti-proliferative effect of celecoxib on CGCCA cells, the treated cells were analysed by flow cytometry to determine the number of cells at each stage of the cell cycle. As shown in Figure 2A and Figure 2B, 1-day treatment with 12, 25, and 50 µM celecoxib increased the proportion of cells at the G2/M phase from 16.32% to 18.04%, 22.59%, and 30.61%, respectively. Most of the cells treated with 100 µM celecoxib were not viable after 2 days of treatment; thus, the distribution of cells at each stage could not be calculated at this concentration (data not shown).

To verify cell cycle arrest in the G2 or M phase, PI staining was performed to identify the M-phase cells with condensed chromatin. As shown in Figure 2C and D, the percentage of M-phase cells did not differ between the control and treated groups, indicating that cell cycle arrest induced by celecoxib in CGCCA cells occurs at the G2 phase rather than at the M phase.

To clarify the possible mechanisms underlying this G2 arrest, we examined Cdc25C, functioning to remove the inhibitory phosphorylation of cyclin dependent kinase (CDKs), and Cyclin dependent kinase 1(CDK-1), two important factors to proceed cell cycle progression from G2 to M. [25], [26] As shown in Figure 2E, celecoxib induced significant dose-dependent repression of CDK-1 expression in CGCCA cells. As for Cdc25C, it seems no significant change was induced in CGCCA cells after celecoxib treatment.

### Apoptosis Analysis

**Figure 3:**
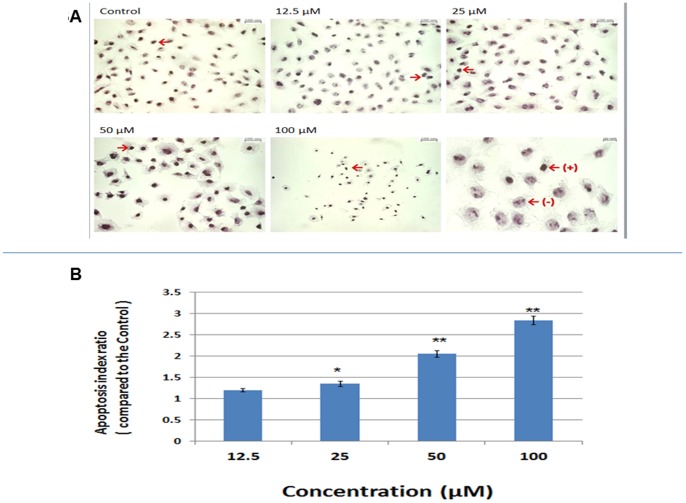
Celecoxib induced apoptosis in CGCCA cells. CGCCA cells were treated with the indicated concentrations of celecoxib for 2 days. (A) TUNNEL assay was used to identify apoptotic cells under different concentrations of celecoxib. Cell morphology and the nuclei were revealed by Harris modified hematoxylin solution counter-staining. The arrows indicated cells positive (+) for TUNNEL assay. The examples of positive (+) and negative (–) cells for TUNNEL assay were shown in the right lower corner panel (B) The apoptotic index of CGCCA cells treated with different concentrations of celecoxib. Each value is a mean ± SD of 3 to 5 determinations. *p<0.05, **p<0.001 (versus control).

Previously, celecoxib was demonstrated to induce apoptosis in CCA [14], [17], [27], [28]. To verify the ability of celecoxib to induce apoptosis in CGCCA cells, we treated CGCCA cells with celecoxib at 12.5, 25, 50, and 100 µM for 2 days. A TUNEL assay was then conducted to calculate the percentage of apoptotic cells. As shown in Figure 3A and B, celecoxib induced CGCCA cells to undergo apoptosis in a concentration-dependent manner.

### The Effect of Celecoxib on Cholangiocarcinoma in vivo

#### (A) Evaluation of the anti-tumour effect of celecoxib on CCA in vivo

**Figure 4:**
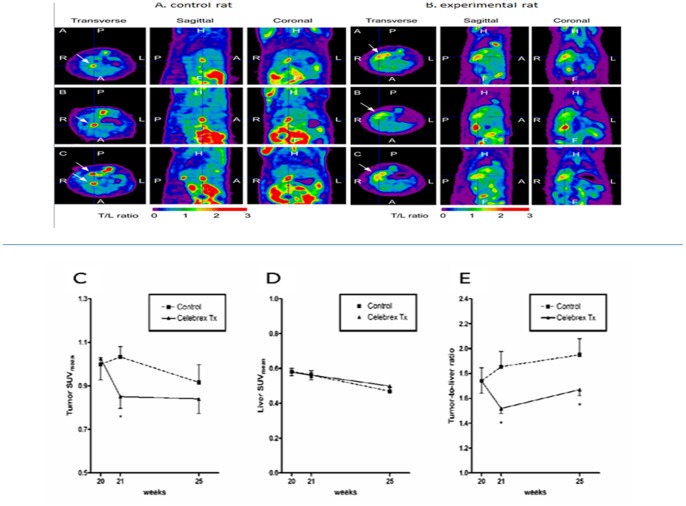
Detection of rat CCA by animal PET and SUV of Tumor, Liver, and Tumor/Liver ratio. (A). Transverse, sagittal, and coronal views of fused CT and PET scans of representing control rats revealed CCA expressing areas of the liver in which the ^18^F-FDG uptake was increased from baseline 1 to 5 weeks after the experiment (i.e., weeks 20, 21, and 25). (B) Transverse, sagittal, and coronal views of fused CT and PET scans of representing rats treated with celecoxib revealed CCA expressing areas of the liver in which the ^18^F-FDG uptake was slightly increased from baseline1 to 5 weeks after the experiment (i.e., weeks 20, 21, and 25). The arrows indicated the “hottest point” with highest ^18^F-FDG uptake. (C) The tumour SUV_mean_ of control rats was initially elevated (week 21) and then decreased to a lower level at the last scan (week 25); however, the tumour SUV_mean_ of rats treated with celecoxib was decreased initially (week 21) and remained at a constant level until the last scan (week 25). (D) The liver SUV_mean_ decreased gradually in control and treated rats during this experiment. (E) The tumour-to-liver (T/L) ratio of SUV showed trend of elevation until the last scans in the control group. In the treatment group, the T/L ratio of SUV was significantly decreased 1 week after celecoxib treatment (control, 1.85±0.12; celecoxib, 1.52±0.04; p<0.05).

After induction of CCA by TAA, tumours in the control and treated groups were evaluated by animal PET-computed tomography (CT) on transverse, sagittal, and coronal views. As shown in Figure 4A and B, both groups showed at least 1 FDG-avid tumour in the liver after 20 weeks of TAA treatment. In the experimental groups, rats were given celecoxib at doses of 40, 80, and 160 mg/kg. Rats treated with 40 and 80 mg/kg celecoxib were similar to those in the control group. Thus, we focused on the rats treated with 160 mg/kg celecoxib. The SUV values of tumours and livers and the T/L ratios for both groups are shown in Figure 4C–E. The celecoxib-treated group clearly showed a lower T/L ratio at 21 and 25 weeks than the control group (Figure 4E) (p<0.05). Accordingly, 160 mg/kg celecoxib treatment resulted in partial but significant suppression of the tumourous growth of rat CCA in vivo.

#### (B) Celecoxib induced apoptosis in CCA tissues in vivo

**Figure 5:**
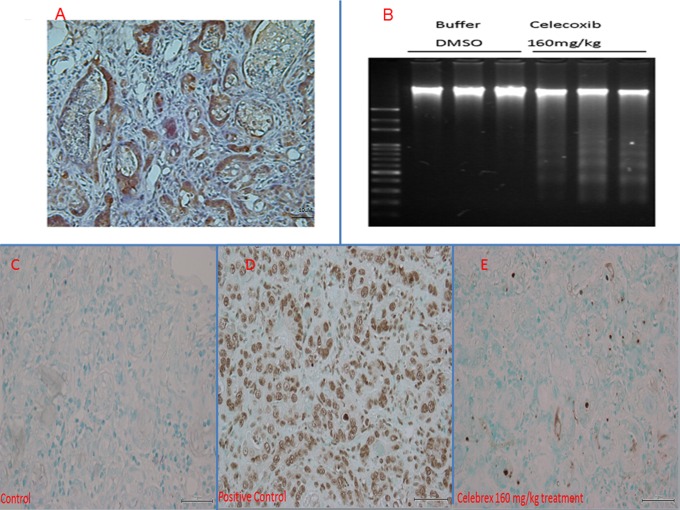
COX-2 expression in rat CCA and apoptosis detection in treated rat CCA. (A) Rat CCA tissue revealed positive cytoplasmic immunoreactivity for COX-2 (×400). (B) Apoptotic DNA fragmentation of rat CCA tissue after treatment with celecoxib (160 mg/kg body weight) for 5 weeks visualised by the DNA laddering assay. No apoptotic DNA fragmentation was observed in the control group treated with DMSO buffer for 5 weeks. (C, D, E) TUNNEL assay for determination of rat CCA apoptosis. The positive cells for TUNNEL assay with brown stains are shown in the positive control (TACS-Nuclease treated tissues ) figure (D). No apoptosis was detected in the control group (C). (E) Apoptosis cells were detected in rat CCA tissues treated with celecoxib (160 mg/kg body weight).

After 25 weeks, the rats were sacrificed and TAA-induced CCA tissues were harvested. After immunohistochemical staining, the rat CCA tissues exhibited prominent cytoplasmic expression of COX-2 (Figure 5A), which agrees with the results shown in Figure 1A. CCA tissues in the group treated with 160 mg/kg celecoxib showed much more prominent DNA fragmentation as than the control group as demonstrated by the DNA laddering assay (Figure 5B). The similar results were obtained as checked the rat CCA tissues by TUNNEL assay (Figure 5C,D, and E).

### COX-2 Expression in Human CCA

COX-2 is diffusely expressed in the cytoplasm in human MF-CCA (Figure S1A–D in File S1). Four representative examples of COX-2 immunohistochemical staining in human CCA were graded from 0 to 3+, with 0 and 1 indicating low expression and 2 and 3 indicating high expression (Figure S1A–D in File S1).

### Clinicodemographic Features and COX-2 Expression Levels in Patients with MF-CCA

44 of 78 specimens (56.4%) obtained from MF-CCA patients revealed high expression of COX-2 (2+ and 3+ positive). Clinicodemographic features were similar between patients with low and high COX-2 expression (Table S1 in File S1).

### Survival and Prognostic Analysis of MF-CCA Patients who Underwent Hepatectomy

A total of 78 MF-CCA patients who had undergone hepatectomy were enrolled in the survival analysis study. The follow-up duration ranged from 1.4 to 94.1 months (median = 13.6 months). The overall survival (OS) rates at 1, 3, and 5 years were 55.1%, 22.9%, and 14.9%, respectively (data not shown). The clinicodemographic data for MF-CCA patients with low or high COX-2 expression is similar (Table S1 in File S1).

Univariate log-rank analysis identified the following factors as adverse influences on the OS rate of the 78 MF-CCA patients who had undergone hepatectomy: presence of symptoms, high preoperative alkaline phosphatase and carcinoembryonic antigen levels, tumour size >5 cm, high COX-2 expression, and positive surgical margin status (Table S2 in File S1). After examining these factors using multivariate Cox proportional hazard analysis, only negative margin status and low COX-2 expression independently predicted favourable OS for MF-CCA patients after hepatectomy (Table 1).

**Table 1:** Cox’s Proportional Hazards Analysis.

Factors	Relative Risk (95% confidence interval)	*p*
Symptoms (positive/negative)	1.408(0.284−86.983)	0.676
ALP(**≦**94 g/dl/>94g/dl)	1.167(0.496−2.750)	0.723
Tumor size (>5 cm/**≦**5 cm)	1.851(0.888−3.857)	0.100
Margin(positive/negative)	3.569(1.622−7.856)	**0.002**
Serum CEA (>5 ng/dl/**≦**5 ng/dl)	0.687(0.327−1.444)	0.322
COX2 expression(high/low)	10.373(3.992−26.956)	**<0.001**

ALP: alkaline phosphatase; CEA: carcinoembryonal antigen.

## Discussion

CCA, which develops from the epithelial lining of the biliary tract, is a lethal disease that yearly affects many thousands worldwide. CCA accounts for 10–15% of hepatobiliary neoplasms, and its incidence and mortality have been recently increasing [4], [5], [29]. Surgical resection provides the best chance of a cure for patients with this disease. For resectable CCA, the 5-year survival rate is approximately 22–44% after radical resection [30], [31]. However, only one-third of CCA patients present with a resectable tumour at the time of diagnosis [32], [33]. Currently, there is no effective chemotherapy regimen for CCA that benefits survival. The current recommended regimen is a combination of cisplatin and gemcitabine as suggested in the UK NCRI ABC-02 trial [34]. Radiotherapy plays a role in palliation for advanced CCA [35], [36]. Thus, the development of a new therapeutic regimen for CCA patients is urgently needed.

Overexpression of COX-2 and subsequent overproduction of prostaglandins have been implicated in variety of neoplastic diseases including pancreas, breast, liver, lung, and other cancers [37]–[39]. Regarding CCA, COX-2 is expressed abundantly in cancerous bile ducts but not in normal bile ducts [12], [16]. Moreover, because COX-2 and its corresponding prostaglandins, mainly PGE2, are able to induce cell proliferation and promote cell survival [39]–[41], targeting COX-2 may effectively aid in the treatment of CCA.

As shown in Figures 1A, 5A, and Fig. S1 in File S1, COX-2 is strongly expressed in CGCCA cells, TAA-induced rat CCA tissue, and human CCA tissues based on immunohistological analysis. The selective COX-2 inhibitor, celecoxib, exhibited a concentration- and time-dependent anti-proliferative effect on CGCCA cells (Figure 1B–D). However, the COX-2 expression in CGCCA cells after celecoxib treatment was not measured in the current study. To further understand the mechanisms by which celecoxib represses CGCCA cell growth, we analysed the distribution of CGCCA cells at various stages of the cell cycle after celecoxib treatment. Previously, celecoxib was shown to induce cell cycle arrest at the G1/S phase through upregulation of the cyclin-dependent kinase inhibitors p21 and p27 [42]. In this report, we found that 1 day of celecoxib treatment induced cell cycle arrest at the G2/M phase rather than the G1/S phase in CGCCA cells (Figure 2A and B). Because G2 and M phases cannot be differentiated by flow cytometry with PI staining because of similar amounts of DNA, we counted M-phase cells, which have condensed chromatins, after PI staining microscopically. As shown in Figure 2C and D, the number of cells in the M phase did not increase with celecoxib treatment, indicating that celecoxib-induced cell cycle arrest occurred at the G2 phase in CGCCA cells. We also examined the presumed target proteins of celecoxib, p21, and p27, in CGCCA cells after celecoxib treatment but found no difference in their expression (data not shown). Taken together, our data indicate that celecoxib induces cell cycle arrest at the G2 phase rather than at the G1 phase in CGCCA cells, indicating that celecoxib works in a highly cell-specific manner.

During cell cycle progression, the combination of specific cyclins and cyclin dependent kinases (CDKs) plays a critical role. Among these, CDK-1 is crucial for G2/M progression [25], [26]. The activation of cyclin-bound CDK-1 requires the removal of inhibitory phosphorylation by Cdc25C [25]. As shown in Figure 2E, celecoxib treatment induced a dose-dependent decrease in CDK1 expression without significantly downregulating Cdc25C expression. Taken together, our data indicate that celecoxib induces downregulation of CDK-1 in CGCCA cells, which could lead to G2 arrest.

In addition to disturbance of cell cycle progression, COX-2 inhibition has been shown to induce apoptosis in CCA tissues through the inactivation of Akt [14], [17], [27], [28]. Zhang et al. further demonstrated that COX-2 inhibition also induced Bax translocation in mitochondria, which resulted in release of cytochrome c from the mitochondria, thus activating caspase 3 and 9, which are involved in the intrinsic apoptotic pathway [17]. Other COX-2-related apoptosis-inducing mechanisms include repression of PDK1 and PTEN [43]–[45]. In agreement with previous reports, our current data also showed that celecoxib induced CGCCA cell apoptosis in vitro in a concentration-dependent manner after 3 days of treatment as determined by the TUNEL assay (Figure 3A and B).

Although numerous studies have demonstrated the anti-tumour effect of COX-2 inhibitors, their clinical application is impeded because of the severe cardiovascular side effects that occur during long-term administration for chemoprevention [18], [46], [47]. To investigate the effectiveness of short-term use of COX-2 inhibitors for the treatment of CCA, we used the previously established TAA-induced rat CCA model [19]. This model recapitulates the histological progression of human CCA [20], indicating that it is a good platform to investigate new CCA treatment regimens. As shown in Figure 5A, TAA-induced CCA presented increased COX-2 expression. Animal PET was then used to measure tumour response to celecoxib treatment [21]. Because lesions <2 mm in size could not be detected on our animal PET and the border of invasive CCA was indistinguishable from the normal liver background, the T/L ratio of SUV was used to represent tumour growth [21].

The experimental rats were dosed with 40, 80, and 160 mg/kg of celecoxib for 5 weeks as indicated in Materials and Methods. The results for rats treated with 40 and 80 mg/kg celecoxib were similar to those of the control group (data not shown). The T/L ratio of SUV was significantly lower in rats treated with 160 mg/kg celecoxib than in rats of the control group at different time points (Figure 4E), suggesting that tumour growth was repressed in rats treated with 160 mg/kg celecoxib. The CCA in group treated by 160 mg/kg celecoxib showed prominent DNA fragmentation (Figure 5B), indicating celecoxib repressed tumour growth through apoptosis induction in vivo, which is also supported by TUNNEL assay results for rat CCA tissues (Figure 5C, D, and E). Although the use of celecoxib for chemoprevention induces severe cardiovascular side effects [18], [46], [47], during the experimental period, no obvious observable side effects were noted in the treated rats. However, the clinical application of short-term high dose of celecoxib still needs more studies to confirm its safety.

Although COX-2 overexpression in CCA has been demonstrated in previous studies [12], [16], [48], the relationship between COX-2 expression and CCA survival remains unclear. Schmitz et al. demonstrated that COX-2 expression is associated with poor prognosis of intrahepatic CCA after hepatectomy. Additionally, COX-2 expression was also linked with lower proliferation and high apoptosis as determined by Ki67 expression and TUNEL assay through immunohistochemistry [49]. As shown in Table 1, 56.4% of MF-CCA patients presented with high COX-2 expression, and the clinical demographic data of MF-CCA patients with high and low COX-2 expression is similar. After hepatectomy, only resection margin status and COX-2 expression level influenced the OS of MS-CCA independently (Table 1).

In conclusion, CCA is a devastating disease with a very dismal outcome despite available chemotherapy and radiotherapy. Radical surgery is the only way to improve survival, but it is often not feasible because of late diagnosis. COX-2 is deemed an oncogene in a variety of cancers, and COX-2 is frequently overexpressed in CCA. The clinical application of COX-2 inhibitors is limited to their negative cardiovascular side effects. In this study, we demonstrated that celecoxib inhibited CCA growth in vivo and in vitro without causing obvious observable side effects. The strong correlation between high COX-2 expression and poor survival of MF-CCA patients further justifies the need for a COX-2 inhibitor for the treatment of CCA. On the basis of our results, we conclude that a short-term high dose of celecoxib may be a promising therapeutic strategy for CCA treatment. Further studies are warranted given the lack of effective treatment for CCA and its dismal prognosis.

## Supporting Information

File S1 **This file contains a supporting figure and supporting tables.** Figure S1, COX-2 is diffusely expressed in the cytoplasm in human MF-CCA. Table S1, Clinicopathological features between COX II high expression and low expression of mass-forming CCA patients underwent hepatectomy. Table S2, Univariate Analysis of Factors Influencing the Overall Survival of the 78 MF-CCA Patients
(DOC)
