# Supplementary material for: Reappraisal of the Therapeutic Role of Celecoxib in Cholangiocarcinoma
Source: PLoS One. 2013 Jul 26;8(7):e69928. doi: 10.1371/journal.pone.0069928 (PMC3724720; doi:10.1371/journal.pone.0069928)
Supplement: File S1 — This file contains a supporting figure and supporting tables. Figure S1, COX-2 is diffusely expressed in the cytoplasm in human MF-CCA. Table S1, Clinicopathological features between COX II high expression and low expression of mass-forming CCA patients underwent hepatectomy. Table S2, Univariate Analysis of Factors Influencing the Overall Survival of the 78 MF-CCA Patients (DOC) [file pone.0069928.s001.doc]

| Table S1 Clinicopathological features between COX II high expression and low expression of mass-forming CCA patients underwent hepatectomy | | | |
| --- | --- | --- | --- |
|  | COX II  low expression (n=34) | COX II  high expression (n=44) | *p* |
| Age (years) | 57.8±13.1 | 61.2±11.0 | 0.212 |
| Gender |  |  | .455 |
| Male | 16(48.5%) | 17(51.5%) |  |
| Female | 18(40.0%) | 27(60.0%) |  |
| Symptom |  |  | .080 |
| Positive | 8(66.7%) | 4(33.3%) |  |
| Negative | 26(39.4%) | 40(60.6%) |  |
| AST (IU/l) |  |  | .414 |
| ≦34 | 19(48.7%) | 20(51.3%) |  |
| >34 | 15(39.5%) | 23(60.5%) |  |
| ALT (U/L) |  |  | .640 |
| ≦36 | 19(47.5%) | 21(52.5%) |  |
| >36 | 13(41.9%) | 18(58.1%) |  |
| ALP (U/L) |  |  | .146 |
| ≦94 | 13(56.5%) | 10(43.5%) |  |
| >94 | 20(38.5%) | 32(61.5%) |  |
| Bilirubin (total) (mg/dl) |  |  | .951 |
| ≦1.3 | 28(43.8%) | 36(56.3%) |  |
| >1.3 | 6(42.9%) | 8(57.1%) |  |
| Albumin (g/dl) |  |  | .323 |
| ≦3.5 | 8(36.4%) | 14(63.6%) |  |
| >3.5 | 24(49.0%) | 25(51.0%) |  |
| Serum CEA (ng/ml) |  |  | .021^*^ |
| ≦5 | 14(56.0%) | 11(44.0%) |  |
| >5 | 8(25.8%) | 23(74.2%) |  |
| Size (cm) |  |  | .484 |
| ≦5 | 16(50.0%) | 16(50.0%) |  |
| >5 | 18(41.9%) | 25(58.1%) |  |
| Lymph node |  |  | .217 |
| Negative | 23(48.9%) | 24(51.1%) |  |
| Positive | 10(34.5%) | 19(65.5%) |  |
| Differentiated |  |  | .261 |
| Well | 0(0.0%) | 2(100.0%) |  |
| Moderate | 15(36.6%) | 26(63.4%) |  |
| Poorly | 18(54.5%) | 15(45.5%) |  |
| other | 1(50.0%) | 1(50.0%) |  |
| Margin |  |  | .087 |
| Negative | 27(50.0%) | 27(50.0%) |  |
| positive | 7(29.2%) | 17(70.8%) |  |
| Post Chemotherapy |  |  | .522 |
| Without (n=33) | 13(39.4%) | 20(60.6%) |  |
| With (n=45) | 21(46.7%) | 24(53.3%) |  |
| Post Radiotherapy |  |  | .806 |
| Without (n=68) | 30(44.1%) | 38(55.9%) |  |
| With (n=10) | 4(40.0%) | 6(60.0%) |  |

| **Supplementary Table 2 Univariate Analysis of Factors Influencing the Overall Survival of the 78 MF-CCA Patients** | | | | | |
| --- | --- | --- | --- | --- | --- |
| Factors | Survival Time (months) | | | | *p* |
|  | Median | 95% CI of Median | 3-year (%) | 5-year (%) |  |
| Gender |  |  |  |  | 0.719 |
| Male (n=33) | 14.70 | 7.89-21.50 | 21.2 | 18.2 |  |
| Female (n=45) | 10.82 | 5.85-15.79 | 24.4 | 15.0 |  |
| Age |  |  |  |  | 0.505 |
| ≦60 (n=38) | 12.89 | 4.45-21.33 | 26.3 | 21.1 |  |
| >60 (n=40) | 12.99 | 7.23-18.74 | 20.0 | 12.0 |  |
| Symptoms |  |  |  |  | **0.006** |
| Negative (n=12) | 37.71 | 4.78-70.63 | 58.3 | 41.7 |  |
| Positive (n=66) | 10.45 | 5.84-15.07 | 16.7 | 11.9 |  |
| AST (IU/l) |  |  |  |  | 0.197 |
| **≦**34 (n=39) | 13.32 | 9.53-17.10 | 30.8 | 22.8 |  |
| >34 (n=38) | 10.72 | 2.28-19.16 | 15.8 | 10.5 |  |
| ALT (IU/l) |  |  |  |  | 0.625 |
| **≦**36 (n=40) | 12.99 | 6.41-19.56 | 25.0 | 19.7 |  |
| >36 (n=31) | 14.70 | 7.81-21.58 | 19.4 | 9.7 |  |
| ALP (IU/L) |  |  |  |  | **0.009** |
| **≦**94 (n=23) | 23.90 | 11.65-36.15 | 39.1 | 29.8 |  |
| >94 (n=52) | 9.11 | 4.92-13.29 | 15.4 | 5.0 |  |
| Bil (total) (mg/dl) |  |  |  |  | 0.581 |
| **≦**1.3 (n=64) | 12.99 | 6.41-19.56 | 25.0 | 16.9 |  |
| >1.3 (n=14) | 10.72 | 0.00-22.23 | 14.3 | 14.3 |  |
| Albumin (g/dl) |  |  |  |  | 0.063 |
| **≦**3.5 (n=22) | 4.70 | 3.11-6.29 | 18.2 | 13.6 |  |
| >3.5 (n=49) | 19.04 | 13.31-24.76 | 24.5 | 15.9 |  |
| Serum CEA (ng/dl) |  |  |  |  | **0.043** |
| **≦**5 (n=25) | 18.51 | 2.09-34.93 | 40.0 | 27.0 |  |
| >5 (n=31) | 10.29 | 4.05-16.53 | 6.5 | 6.5 |  |
| Margin |  |  |  |  | **<0.001** |
| Negative (n=54) | 19.43 | 14.97-23.89 | 33.3 | 23.8 |  |
| Positive (n=24) | 4.41 | 2.43-6.38 | 0.0 | 0.0 |  |
| Size |  |  |  |  | **0.006** |
| **≦**5cm (n=32) | 19.99 | 13.75-26.23 | 37.5 | 30.7 |  |
| >5cm (n=43) | 9.11 | 1.97-16.25 | 14.0 | 7.0 |  |
| Lymph node |  |  |  |  | 0.063 |
| Negative (n=47) | 19.89 | 16.71-23.07 | 29.8 | 18.7 |  |
| Positive (n=29) | 10.45 | 0.00-22.71 | 13.8 | 13.8 |  |
| Histology |  |  |  |  | 0.207 |
| Well (n=2) | 2.73 | NA | 0.0 | 0.0 |  |
| Moderate (n=41) | 13.84 | 7.45-20.23 | 22.0 | 17.1 |  |
| Poor (n=33) | 12.99 | 5.25-20.72 | 27.3 | 17.3 |  |
| Others(n=2) | 4.37 | NA | 0.0 | 0.0 |  |
| COX II |  |  |  |  | **<0.001** |
| Low expression (n=34) | 30.28 | 13.51-47.05 | 47.1 | 34.8 |  |
| High expression (n=44) | 5.79 | 3.29-8.28 | 4.5 | 2.3 |  |
| Post-op Chemotherapy |  |  |  |  | 0.937 |
| Without (n=33) | 5.65 | 2.47-8.84 | 30.3 | 23.9 |  |
| With (n=45) | 14.70 | 9.64-19.75 | 17.8 | 11.1 |  |
| Post-op Radiotherapy |  |  |  |  | 0.071 |
| Without (n=68) | 13.32 | 8.27-18.36 | 26.5 | 18.9 |  |
| With (n=10) | 6.97 | 5.29-8.65 | 0.0 | 0.0 |  |
| MF: mass-forming; AST: aspartate aminotransferase; ALT: alanine amionotransferase; ALP: alkaline phosphatase; CEA: carcinoembryonal antigen; CA 19-9: carbohydrate antigen; IU: international unit; op: operation | | | | | |

**Figure 1. COX-2 is diffusely expressed in the cytoplasm in human MF-CCA.** Four representative samples of COX-2 immunohistochemical staining in human CCA from negative to 3+ (A, B, C, D: ×400).
